# Spleen stiffness measurement predicts decompensation and rules out high-risk oesophageal varices in primary biliary cholangitis

**DOI:** 10.1016/j.jhepr.2023.100952

**Published:** 2023-10-31

**Authors:** Cristina Rigamonti, Micol Giulia Cittone, Giulia Francesca Manfredi, Carla De Benedittis, Noemi Paggi, Francesca Baorda, Davide Di Benedetto, Rosalba Minisini, Mario Pirisi

**Affiliations:** 1 Department of Translational Medicine, Università del Piemonte Orientale, Novara, Italy; 2 Division of Internal Medicine, AOU Maggiore della Carità, Novara, Italy

**Keywords:** Vibration-controlled transient elastography, Portal hypertension, Liver stiffness, Outcome

## Abstract

**Background & Aims:**

Primary biliary cholangitis (PBC) may lead to portal hypertension (PH). Spleen stiffness measurement (SSM) by vibration-controlled transient elastography accurately predicts PH. We aimed to assess SSM role in stratifying the risk of liver decompensation in PBC.

**Methods:**

In this monocentric, prospective, cross-sectional study, we included 114 patients with PBC who underwent liver stiffness measurement (LSM) and SSM. In total, 78 and 33 patients underwent two and three sequential vibration-controlled transient elastography examinations, respectively (longitudinal study). Screening for high-risk oesophageal varices by oesophagogastroduodenoscopy was performed according to guidelines and proposed to all patients with SSM >40 kPa.

**Results:**

Among the 114 patients, 20 (17%) had LSM ≥10 kPa, whereas 17 (15%) had SSM >40 kPa. None of the patients with SSM ≤40 kPa had high-risk oesophageal varices, compared with three of 14 patients with SSM >40 kPa (21%; three refused endoscopy); any-size oesophageal varices were found in nine of 14 patients (64%). During a median follow-up of 15 months (IQR 10–31 months), five (4%) patients developed liver decompensation. The probability of liver decompensation was significantly higher among patients with both LSM ≥10 kPa and SSM >40 kPa: 41% at 24 months *vs*. 0% in other patient groups (*i.e.* LSM <10 kPa and SSM ≤40 kPa, or LSM ≥10 kPa and SSM ≤40 kPa, or LSM <10 kPa and SSM >40 kPa) (*p* <0.0001). Among the 78 patients undergoing longitudinal evaluation, four of nine patients (44%) with SSM increase during follow-up experienced liver decompensation, whereas none of those with stable LSM and SSM had liver decompensation.

**Conclusions:**

Both LSM and SSM predict liver decompensation in patients with PBC. SSM ≤40 kPa rules out high-risk oesophageal varices and might be used in combination with LSM to improve the prediction of PH-related complications.

**Impact and implications:**

Spleen stiffness measurement by vibration-controlled transient elastography accurately predicts portal hypertension in patients with chronic viral hepatitis. The present study is the first to demonstrate that in primary biliary cholangitis the combination of liver stiffness and spleen stiffness measurement can significantly improve risk stratification by predicting liver decompensation. Moreover, when spleen stiffness is combined with liver stiffness measurement and platelet count, it aids in identifying individuals with a low probability of having high-risk oesophageal varices, thereby allowing the avoidance of unnecessary endoscopy examinations. Further validation of our results in larger cohorts of patients with primary biliary cholangitis is needed to implement spleen stiffness measurement in clinical practice.

## Highlights

• Combination of LSM <10 kPa and SSM ≤40 kPa identified patients with PBC not at risk of liver decompensation with 100% sensitivity.
• SSM ≤40 kPa ruled out high-risk oesophageal varices in PBC.
• By applying Baveno VII criteria in our cohort of patients with PBC, 41.7% of endoscopies would have been correctly saved.
• Changes over time in both LSM and SSM by VCTE might predict progression of liver disease in patients with PBC.

## Graphical abstract

**Figure:**
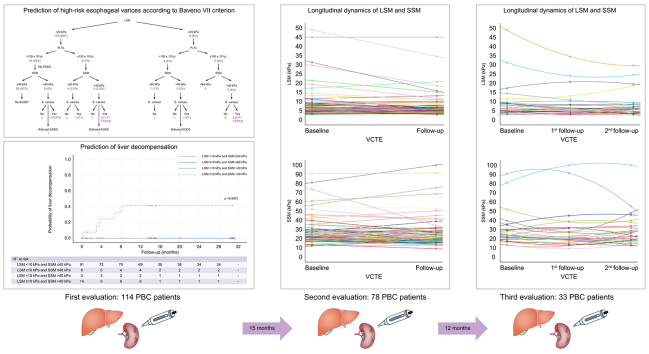

## Introduction

Primary biliary cholangitis (PBC) is a chronic autoimmune cholangiopathy^1^ that predominantly affects women,^2^^,^^3^ leading to immune-mediated damage to biliary ducts.^4^^,^^5^ Even though the disease typically progresses slowly and has a benign course, untreated PBC can result in severe liver fibrosis, cirrhosis, and associated complications.^6^ Although patients achieving biochemical response to treatment with ursodesoxycholic acid alone or in combination with obeticholic acid have estimated survival rates of approximately 80% at 10 years,[7], [8], [9], [10] advanced fibrosis remains an independent risk factor for poor outcome. Hence, the prediction of fibrosis is crucial for both diagnostic and follow-up purposes.^11^

Liver stiffness measurement (LSM) by vibration-controlled transient elastography (VCTE) has emerged as one of the most reliable surrogate methods for detecting severe fibrosis among patients with PBC.[12], [13], [14] LSM by VCTE is recommended at baseline to differentiate between early and advanced stage disease (cut-off value ≥10 kPa) and might be repeated during treatment for risk stratification.^15^ Recently, a large multicentre retrospective study involving 3,985 patients with PBC demonstrated that LSM assessed by VCTE improves survival prediction beyond biochemical response, established prognostic scores, and age categories, regardless of time.^16^

Progression of liver damage in PBC can lead to portal hypertension (PH), which can affect up to 10% of patients in a precirrhotic stage, mainly caused by a presinusoidal component.^17^^,^^18^ Early identification of patients with clinically significant portal hypertension (CSPH) is crucial to prevent complications such as variceal bleeding. Although hepatic venous pressure measurement (HVPG) remains the gold standard, VCTE plays an important role as a non-invasive and widely available alternative, as LSM also correlates with portal pressure.^19^^,^^20^

More recently, spleen stiffness measurement (SSM) has been introduced as a complementary tool to LSM for the non-invasive prediction of CSPH. Several studies have shown that SSM by VCTE correlates with the severity of PH.[21], [22], [23] Furthermore, SSM by VCTE has proven to be a highly reproducible and easily applicable technique in patients with chronic liver disease.^24^ Based on this evidence, Baveno VII guidelines have proposed using SSM ≤40 kPa in patients with compensated advanced chronic liver disease (cACLD) caused by viral hepatitis in whom endoscopy would be required according to the Baveno VI criteria (LSM ≥20 kPa or a platelet count ≤150 × 10^3^/μl) to avoid oesophagogastroduodenoscopy (EGDS), as this SSM cut-off is associated with a low probability of high-risk varices.^25^^,^^26^ However, further investigation is required to determine the applicability of this criterion for cACLD caused by other aetiologies, including autoimmune and cholestatic diseases.

To the best of our knowledge, no studies have investigated clinical significance of SSM in the context of PBC. SSM could potentially provide additional data in PBC caused by the well-known presence of a presinusoidal component of PH in this setting. Therefore, the aim of this single-centre study was to investigate the prognostic value of SSM in stratifying the risk of liver decompensation and to explore its ability to rule out high-risk varices among patients affected by PBC. In addition, we aimed to assess the longitudinal dynamics of SSM during follow-up and determine their clinical significance in PBC.

## Patients and methods

### Patients

In this single-centre, prospective, cross-sectional and longitudinal study, all consecutive patients with an established diagnosis of PBC according to European guidelines and a follow-up in a tertiary referral centre for liver diseases (Internal Medicine Department – Novara) between September 2020 and April 2023,^7^ were included. Exclusion criteria were a lack of informed consent and failure of VCTE examination.

Demographic and clinical features at baseline and at the time of VCTE examination, development of liver decompensation (*i.e.* gastrointestinal bleeding, ascites, and hepatic encephalopathy), liver transplantation, or death during follow-up were recorded.

All patients underwent screening for varices with EGDS according to Baveno VI guidelines (LSM ≥20 kPa and platelets ≤150 × 10^3^/μl).^25^^,^^26^ EGDS was also proposed to all patients with SSM >40 kPa, independently of LSM and platelet count.

The study was conducted in full accordance with Helsinki criteria and has been approved by the local ethical committee.

### Vibration-controlled transient elastography

VCTE examinations for LSM and SSM were performed using the FibroScan® 630 Expert machine (Echosens, Paris, France), equipped with liver-dedicated (LSM@50Hz) and spleen-dedicated (SSM@100Hz) modules coupled with an ultrasound localisation system for the spleen. Results were expressed in kPa. LSM and SSM were considered reliable only if at least 10 successful measurements were obtained, with a success rate of at least 60%, and the IQR-to-median ratio was ≤0.3. Failure of the examination was defined as absence of any valid measurement.^27^ LSM and SSM were performed by placing the patient in a supine position with the right and left arms, respectively, in maximum abduction and by placing the transducer in the right and left intercostal spaces, respectively. For SSM, the tip of the probe transducer was placed in a previously ultrasound-targeted point in which the spleen parenchyma had been previously identified.

Overall, 115 patients with PBC underwent at least one VCTE examination (cross-sectional study); 78 and 33 patients underwent two and three sequential examinations for LSM and SSM, respectively, at least 12 months apart (longitudinal study). The time frame for repeat VCTE examinations was defined according to guidelines.^15^

For patients who underwent longitudinal VCTE measurement, changes in LSM and SSM during follow-up were defined as significant for ≥30% variation of the baseline value. The 30% threshold of LSM/SSM changes corresponds to the accepted variability of VCTE assay.

### Diagnostic criteria

The diagnosis of cirrhosis was defined according to previous liver histology (previous liver biopsy when available), imaging (ultrasound or computed tomography or magnetic resonance imaging) compatible with cirrhosis, and clinical or laboratory data.^28^ Splenomegaly was defined as spleen longitudinal diameter longer than 12.5 cm. Liver decompensation was defined as the onset of ascites, variceal bleeding, hepatic encephalopathy, or jaundice (serum total bilirubin >3 mg/dl).

The following cut-offs have been used: LSM ≥10 kPa for defining cACLD and SSM ≤40 kPa for low probability of high-risk oesophageal varices.^25^^,^^26^

### Statistical analysis

Statistical analysis was performed using the software SPSS Statistics version 20.0 (IBM Corporation, Armonk, NY, USA). Continuous variables were presented as median and IQR. Categorical data were summarised as absolute frequencies and relative percentages. Comparisons between groups (patients with LSM <10 *vs*. ≥10 kPa; patients with SSM ≤40 *vs*. >40 kPa; baseline *vs*. follow-up data in patients who underwent longitudinal evaluation) were made using the Mann–Whitney *U* test for continuous variables and the Fisher exact test for dichotomous variables. The risk of liver decompensation was evaluated by Kaplan–Meier analysis. The follow-up time was calculated from baseline VCTE examination for LSM/SSM and was censored at the date of liver decompensation for patients who decompensated, at the date of death for deceased patients, and at the last follow-up visit for all others.

The operative ability of LSM ≥10 kPa and SSM >40 kPa in predicting liver decompensation and SSM ≤40 kPa in ruling out high-risk varices was evaluated by Bayesian analysis. The statistical significance level considered was <0.05.

## Results

### Patients

Among the 115 initially selected patients with PBC (110 females and 5 males), one was excluded owing to the failure of VTCE examination related to obesity (BMI >35 kg/m^2^). Thus, our cross-sectional study comprised a total of 114 patients, of which 109 (96%) were female. The median age at diagnosis of PBC was 66 years (IQR 54–71 years), and the median disease duration was 66 months (IQR 33–102 months) at the time of inclusion in the study. The key characteristics of the 114 patients at baseline are summarised in Table 1.

**Table 1:** Key demographic and clinical features of 114 patients with PBC at baseline.

Clinical features	Values
Females, n (%)	109 (96)
Age (years), median (IQR)	66 (53–70)
Disease duration (months), median (IQR)	66 (33–102)
Cirrhosis, n (%)	17 (15)
Splenomegaly, n (%)	26 (23)
Longitudinal spleen diameter (cm), median (IQR)	10.3 (9.4–11.9)
BMI (kg/m^2^), median (IQR)	24.6 (22.3–27)
Laboratory tests	
AMA, n (%)	66 (75)
Anti-sp100, n (%)	14 (16)
Anti-gp210, n (%)	8 (9)
IgM (× ULN), median (IQR)	1.4 (0.9–1.7)
AST (× ULN), median (IQR)	0.7 (0.5–0.9)
ALT (× ULN), median (IQR)	0.6 (0.5–0.8)
GGT (× ULN), median (IQR)	1 (0.2–2)
ALP (× ULN), median (IQR)	1 (0.8–1.5)
Total bilirubin (× ULN), median (IQR)	0.6 (0.5–0.9)
Albumin (× LLN), median (IQR)	1.3 (1.2–1.3)
INR, median (IQR)	0.98 (0.93–1.05)
Platelets (× 10^3^/μl), median (IQR)	236 (185–275)
VCTE	
LSM (kPa), median (IQR)	6.4 (4.7–8.8)
Controlled attenuation parameter (dB/m), median (IQR)	240 (210–263)
SSM (kPa), median (IQR)	22.2 (18.9–29.0)

Categorical variables are reported as absolute numbers with percentages in parentheses, whereas continuous variables are reported as medians with IQRs in parentheses. ALP, alkaline phosphatase; ALT, alanine aminotransferase; AMA, anti-mitochondrial antibodies; Anti-gp210, anti-glycoprotein 210 antibodies; Anti-Sp100, anti-nuclear sp100 antibodies; AST, aspartate aminotransferase; GGT, gamma-glutamyl transferase; INR, international normalised ratio; LLN, lower limit of normal; LSM, liver stiffness measurement; PBC, primary biliary cholangitis; SSM, spleen stiffness measurement; ULN, upper limit of normal; VCTE, vibration-controlled transient elastography.

### Vibration-controlled transient elastography

#### Cross-sectional study

Median LSM was 6.4 kPa (IQR 4.7–8.8 kPa), being ≥10 kPa in 20 (17%) patients. Median SSM was 22.2 kPa (IQR 19–29 kPa), resulting >40 kPa in 17 (15%) patients.

Clinical features of the 114 patients stratified by LSM and SSM are shown in Table 2.

**Table 2:** Comparison of demographic and clinical characteristics of the 114 patients with PBC at cross-sectional VCTE examination, stratified based on the LSM value (<10 kPa *vs.* ≥10 kPa) and the SSM value (≤40 kPa *vs.* >40 kPa).

Clinical features	LSM			SSM		
	<10 kPa (n = 94)	≥10 kPa (n = 20)	*p*	≤40 kPa (n = 97)	>40 kPa (n = 17)	*p*
Age (years), median (IQR)	61 (53–70)	68 (63–75)	0.003	61 (53–70)	67 (64–75)	0.005
Cirrhosis, n (%)	2 (2)	15 (75)	<0.0001	4 (4)	13 (76)	<0.0001
Splenomegaly, n (%)	15 (16)	11 (55)	0.001	13 (13)	13 (76)	<0.0001
Longitudinal spleen diameter (cm), median (IQR)	10.1 (9.3–11.3)	13.3 (10.1–15.4)	0.004	10 (9.2–11.1)	14 (12.1–15.6)	<0.0001
Endoscopic signs of PH, n (%)	1 (1)	9 (45)	<0.0001	1 (1)	10 (59)	<0.0001
Oesophageal varices (any size), n (%)	1 (1)	7 (35)	<0.0001	1 (1)	7 (41)	<0.0001
Laboratory tests						
ALP (× ULN), median (IQR)	1 (0.7–1.5)	1.1 (0.9–1.9)	0.98	1.0 (0.7–1.5)	1.1 (0.9–2)	0.75
AST (× ULN), median (IQR)	0.7 (0.5–0.9)	0.9 (0.7–1.1)	0.007	0.7 (0.6–0.9)	0.9 (0.6–1.2)	0.03
Total bilirubin (× ULN), median (IQR)	0.5 (0.4–0.7)	0.6 (0.5–0.8)	0.19	0.5 (0.4–0.7)	0.6 (0.5–0.8)	0.03
Albumin (× LLN), median (IQR)	1.3 (1.2–1.3)	1.2 (1.1–1.3)	0.06	1.3 (1.2–1.3)	1.3 (1.2–1.3)	0.59
INR, median (IQR)	0.97 (0.91–1.03)	1.05 (0.97–1.21)	0.004	0.96 (0.92–1.01)	1.1 (1.0–1.21)	<0.0001
Platelets (× 10^3^/μl), median (IQR)	245 (210–289)	165 (128–211)	<0.0001	242 (211–286)	141 (121–179)	<0.0001
VCTE						
LSM (kPa), median (IQR)	—	—		5.9 (4.6–7.7)	19.3 (12.8–34.3)	<0.0001
LSM ≥10 kPa, n (%)	—	—		6 (6)	14 (82)	<0.0001
SSM (kPa), median (IQR)	21 (18.5–26.1)	54.4 (34.4–73.2)	<0.0001	—	—	
SSM >40 kPa, n (%)	3 (2)	14 (70)	<0.0001	—	—	

Categorical variables are reported as absolute numbers with percentages in parentheses, whereas continuous variables are reported as medians with IQRs in parentheses. Comparisons between patients with LSM <10 kPa *vs.* LSM ≥10 kPa and patients with SSM ≤40 kPa *vs.* >40 kPa were made using the Mann–Whitney *U* test for continuous variables and the Fisher exact test for dichotomous variables. Levels of significance: *p* <0.05. ALP, alkaline phosphatase; AST, aspartate aminotransferase; INR, international normalised ratio; LLN, lower limit of normal; LSM, liver stiffness measurement; PBC, primary biliary cholangitis; PH, portal hypertension; SSM, spleen stiffness measurement; ULN, upper limit of normal; VCTE, vibration-controlled transient elastography.

Patients with LSM ≥10 kPa exhibited several significant differences compared with those with LSM <10 kPa. First, the former group was significantly older (*p* = 0.003) and had significantly higher frequency of cirrhosis (*p* <0.0001), splenomegaly, endoscopic signs of PH (*p* <0.0001), and any-size oesophageal varices (*p* <0.0001). In addition, these patients had a significantly lower platelet count (*p* <0.0001) and a significantly higher SSM (*p* <0.0001).

Similarly, the 17 patients with SSM >40 kPa displayed remarkable differences compared with the 94 patients with SSM ≤40 kPa. The former group was significantly older (*p* = 0.005) and had higher frequency of cirrhosis (*p* <0.0001). They also showed a higher prevalence of splenomegaly and endoscopic signs of PH (*p* <0.0001) as well as any-size oesophageal varices (*p* <0.0001). Furthermore, these patients had a significantly lower platelet count (*p* <0.0001) and significantly higher LSM (*p* <0.0001).

##### Prediction of liver decompensation by LSM and SSM

**Fig. 1:**
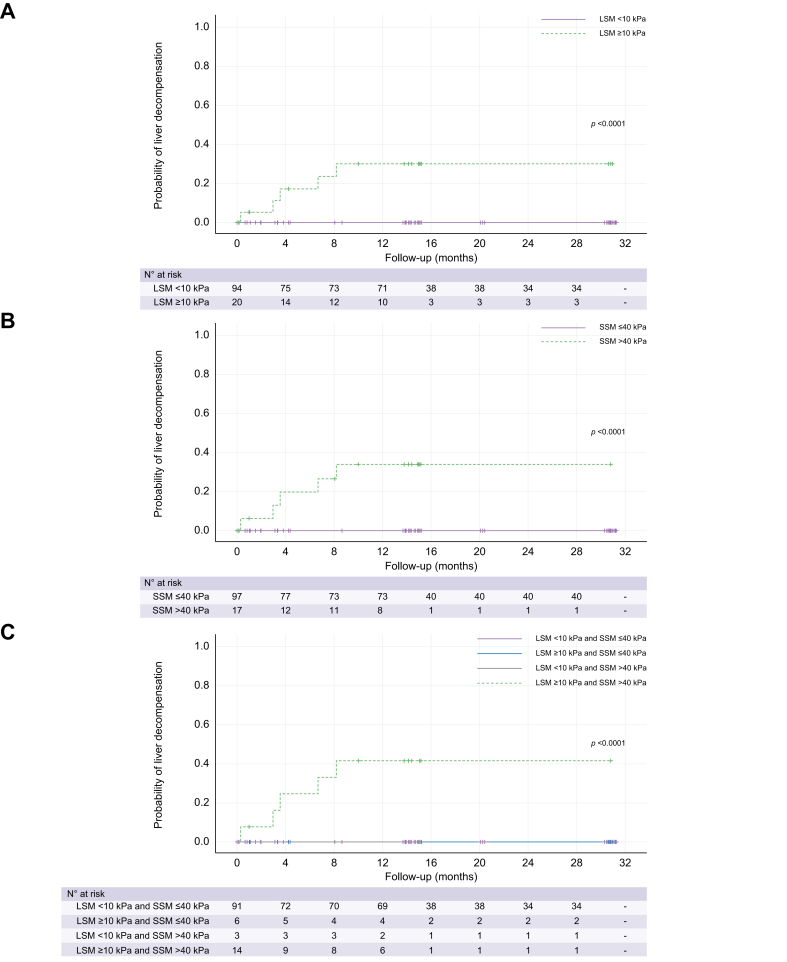
Probability of liver decompensation during the follow-up study period assessed using Kaplan-Meier analysis, stratified by LSM and SSM values. (A) Probability of liver decompensation through the follow-up, based on the LSM value (<10 kPa *vs.* ≥10 kPa) (*p* <0.0001, log-rank test). (B) Probability of liver decompensation through the follow-up based on the SSM value (≤40 kPa *vs.* >40 kPa) (*p* <0.0001, log-rank test). (C) Probability of liver decompensation through the follow-up based on combined LSM and SSM values (LSM<10 kPa and SSM ≤40 kPa *vs.* LSM ≥10 kPa and SSM ≤40 kPa *vs.* LSM<10 kPa and SSM >40 kPa *vs.* LSM ≥10 kPa and SSM ≤40 kPa) (*p* <0.0001, log-rank test). LSM, liver stiffness measurement; SSM, spleen stiffness measurement.

During a median follow-up of 15 months (IQR 10–31 months) following the baseline VCTE examination, liver decompensation occurred in 5 of 114 patients (4%). Three of five patients developed ascites, one patient experienced variceal bleeding, and one patient developed hepatic encephalopathy and jaundice. Stratifying the patients based on their LSM value (LSM <10 kPa *vs*. LSM ≥10 kPa), the probability of liver decompensation (Fig. 1A) was significantly greater among patients with LSM ≥10 kPa (30 *vs*. 0% at 24 months; *p* <0.0001). Similarly, when stratified based on SSM value (SSM ≤40 kPa *vs*. SSM >40 kPa) patients with SSM >40 kPa exhibited a 34% probability of decompensation at 24 months compared with 0% for those with SSM ≤40 kPa (*p* <0.0001), as depicted in Fig. 1B. By combining LSM and SSM, the probability of liver decompensation was significantly higher among patients with both LSM ≥10 kPa and SSM >40 kPa: 41% at 24 months *vs*. 0% in all other groups of patients (*i.e.* those with LSM <10 kPa and SSM ≤40 kPa, those with LSM ≥10 kPa and SSM ≤40 kPa, or those with LSM <10 kPa and SSM >40 kPa; *p* <0.0001) (Fig. 1C). Both LSM ≥10 kPa and SSM >40 kPa demonstrated a sensitivity of 100% in predicting the risk of liver decompensation.

##### Prediction of high-risk oesophageal varices

**Fig. 2:**
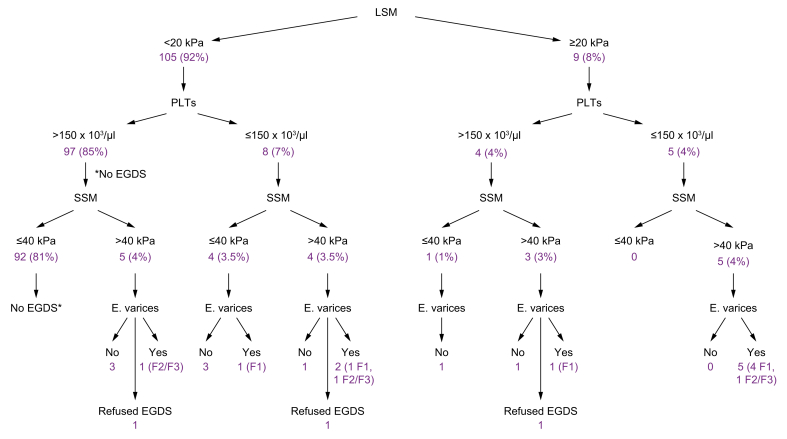
Flow chart of the 114 patients with PBC stratified based on LSM values (<20 *vs.* ≥20 kPa), platelet count (>150 × 10^3^/μl *vs.* ≤150 × 10^3^/μl), and SSM values (≤40 kPa *vs.* >40 kPa) according to Baveno VII Consensus Workshop. For each subgroup, the number of performed endoscopic examinations and the presence of low- or high-risk oesophageal varices, if any, are reported. ∗According to Baveno VI, EGDS was not performed. Fourteen patients underwent EGDS for other reasons, none had any size varices. E.varices, oesophageal varices; EGDS, oesophagogastroduodenoscopy; F1, low-risk varices; F2/F3, high-risk varices; LSM, liver stiffness measurement; PBC, primary biliary cholangitis; PLTs, platelets; SSM, spleen stiffness measurement.

Fig. 2 illustrates the stratification of patients according to the proposed Baveno VI and Baveno VII algorithms. None of the patients with SSM value ≤40 kPa who underwent EGDS exhibited high-risk oesophageal varices, regardless of their LSM value and platelet count. SSM ≤40 kPa demonstrated a sensitivity of 100% in ruling out high-risk varices. Only one patient with SSM 38.4 kPa who underwent EGDS was found to have small oesophageal varices.

Among the 14 patients with SSM >40 kPa who underwent EGDS (excluding three patients who refused endoscopy), 11 (79%) displayed endoscopic signs of PH. Varices of any size were found in nine of 14 patients (64%), and high-risk varices were found in three of 14 patients (21%). One patient with LSM <20 kPa, platelet count >150 × 10^3^/μl, and SSM >40 kPa who underwent EGDS had high-risk varices.

Among the 12 patients with LSM ≥20 kPa or a platelet count ≤150 × 10^3^/μl in whom endoscopy was performed according to the Baveno VI criteria, by applying the proposed Baveno VII algorithm, 41.7% of EGDS would have been correctly saved in those with SSM ≤40 kPa (none of them were found to have high-risk varices at EGDS).

#### Longitudinal study

Seventy-eight patients were included in the longitudinal study: among them, 65 patients with baseline LSM <10 kPa and 13 with baseline LSM ≥10 kPa. Overall, the median time between baseline and the second VCTE examination was 15 months (IQR 12–16 months). In patients with baseline LSM <10 kPa, the median time between the first and second VCTE examinations was 15 months (IQR 12–16 months), and in those with LSM ≥10 kPa, it was 14 months (IQR 12–16 months) (*p* = 0.17).

Among the 33 patients who underwent three sequential VCTE examinations, the median time between the second and third VCTE examinations was 12.5 months (IQR 12.4–12.6 months).

**Fig. 3:**
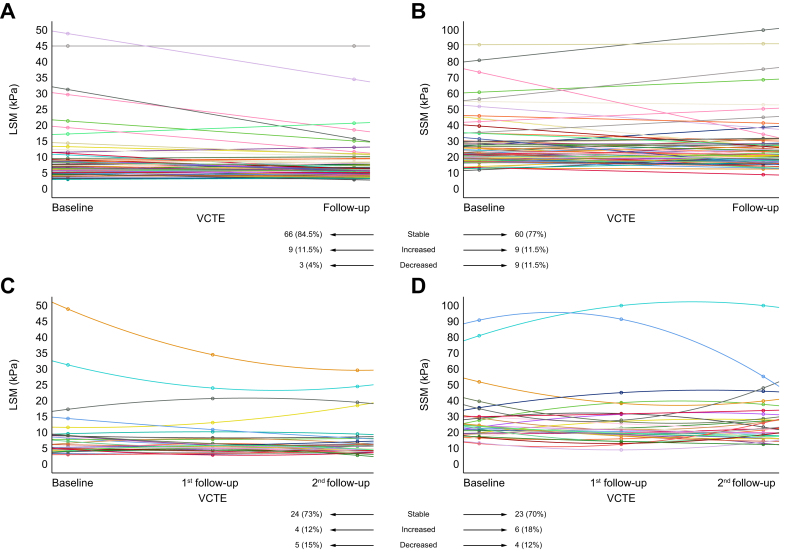
Longitudinal changes in LSM and SSM in the 78 and 33 patients with PBC who underwent two and three sequential VCTE examinations, respectively. Longitudinal changes in (A) LSM and (B) SSM in the 78 patients with PBC who underwent two sequential VCTE examinations. Longitudinal changes in (C) LSM and (D) SSM in the 33 patients with PBC who underwent three sequential VCTE examinations. PBC, primary biliary cholangitis; LSM, liver stiffness measurement; SSM, spleen stiffness measurement; VCTE, vibration-controlled transient elastography.

In the cohort of patients who underwent sequential VCTE evaluation, LSM and SSM did not significantly change during the follow-up (Table 3). The individual dynamic changes of LSM and SSM in patients who underwent two and three sequential VCTE examinations are depicted in Fig. 3.

**Table 3:** Comparison of demographic and clinical characteristics at baseline and follow-up of the 78 patients with PBC who underwent two sequential longitudinal VCTE examinations.

Clinical features	Baseline (n = 78)	Follow-up (n = 78)	*p*
Age (years), median (IQR)	61 (54–68)	62 (55–69)	0.57
Cirrhosis, n (%)	11 (14)	12 (76)	1.0
Splenomegaly, n (%)	19 (24)	20 (26)	1.0
Longitudinal spleen diameter (cm), median (IQR)	10.3 (9.4–11.7)	10.4 (9.1–11.9)	0.70
Endoscopic signs of PH, n (%)	8 (11)	10 (13)	0.76
Oesophageal varices, n (%)	5 (7)	6 (8)	1.0
Laboratory tests			
ALP (× ULN), median (IQR)	0.9 (0.7–1.1)	0.9 (0.7–1.2)	0.94
AST (× ULN), median (IQR)	0.7 (0.6–0.9)	0.9 (0.6–1.2)	0.07
Total bilirubin (× ULN), median (IQR)	0.5 (0.4–0.7)	0.6 (0.4–0.7)	0.97
Albumin (× LLN), median (IQR)	1.3 (1.2–1.3)	1.3 (1.2–1.3)	0.76
INR, median (IQR)	0.98 (0.93–1.04)	1 (0.95–1.05)	0.40
Platelets (× 10^3^/μl), median (IQR)	237 (186–276)	241 (193–290)	0.61
VCTE			
LSM (kPa), median (IQR)	6.1 (4.6–8.6)	6 (14.6–7.9)	0.46
LSM ≥10 kPa, n (%)	13 (17)	13 (17)	1.0
SSM (kPa), median (IQR)	22.1 (18.9–28)	20.9 (17.8–27.9)	0.63
SSM >40 kPa, n (%)	11 (14)	8 (10)	0.62

Categorical variables are reported as absolute numbers with percentages in parentheses, whereas continuous variables are reported as medians with IQRs in parentheses. Comparisons between baseline and follow-up data were made using the Mann–Whitney *U* test for continuous variables and the Fisher exact test for dichotomous variables. Levels of significance: *p* <0.05. ALP, alkaline phosphatase; AST, aspartate aminotransferase; INR, international normalised ratio; LLN, lower limit of normal; LSM, liver stiffness measurement; PBC, primary biliary cholangitis; PH, portal hypertension; SSM, spleen stiffness measurement; ULN, upper limit of normal; VCTE, vibration-controlled transient elastography.

Among the 78 patients who underwent two sequential VCTE examinations (Fig. 3A and B), LSM and SSM remained stable in 84 and 77% of patients, respectively; none of those with stable LSM and SSM experienced liver-related events. However, four of nine patients (44%) with an increase in SSM during follow-up developed liver decompensation. In three patients with an increase in LSM, the value remained below 6.5 kPa, whereas in one patient LSM increased from 9.8 to 13.1 kPa and SSM from 19.8 to 27 kPa. In this last case, follow-up liver biopsy revealed the development of cirrhosis, and endoscopy showed the presence of portal hypertensive gastropathy. Among the two patients in whom both LSM and SSM decreased, one exhibited a decrease from 48 to 33 kPa in LSM and from 52 to 37 kPa in SSM, and subsequent endoscopy demonstrated the disappearance of previously diagnosed small varices. The other patient started ursodesoxycholic acid therapy after the first VCTE examination.

Among the 33 patients who underwent three sequential VCTE examinations (Fig. 3C and D), LSM and SSM remained stable in 73 and 70% of patients, respectively; those with stable LSM and SSM did not experience any liver-related events. However, in one patient, SSM increased from 35 to 51 kPa, and endoscopy revealed progression of varices from small to medium/large size, whereas LSM remained stable.

## Discussion

To our knowledge, this is the first study that evaluated LSM and SSM in a cross-sectional cohort of 114 patients with PBC, with 78 undergoing longitudinal sequential evaluations. Our findings not only reaffirm the prognostic value of LSM in stratifying the risk of hepatic decompensation during follow-up in patients with PBC but also, for the first time, demonstrate the complementary role of SSM in non-invasively predicting PH and decompensation in PBC.

The application of the Baveno VII criteria, originally proposed for viral hepatitis, to our study population has shown that the combination of SSM, LSM, and platelet count significantly enhances the prediction of varices risk. This holds particular significance in the context of PBC, given that a substantial proportion of patients (up to one-third) may present with CSPH in a non-cirrhotic disease stage,^17^^,^^18^^,^^29^ and PH represents the most common cause of decompensation and death.[30], [31], [32]

A study by Warnes *et al.*,^18^ conducted on a cohort of 86 patients with PBC, has shed light on the early development of PH in the disease. Notably, the authors observed that 34% of their patients with PBC in the pre-cirrhotic stage exhibited severe PH (HVPG >12 mmHg), which appeared to be associated with histologic lesions in the portal and sinusoidal tract and predicted a high risk of worse outcome in terms of both survival and liver complications.^32^ This finding underscores the importance of early identifying PH in patients with PBC, even in the absence of cirrhosis, anticipating both an increase in bilirubin level and cirrhosis. However, HVPG used by Warnes *et al.*^18^ to evaluate portal pressure is an invasive procedure with limited availability outside tertiary liver centres.

Recently, VCTE has gained increasing popularity as a good non-invasive method for assessing liver disease severity. Despite its limitations, such as the potential overestimation of fibrosis in the presence of confounders, LSM has demonstrated its value in diagnosing advanced chronic liver disease and in prognostically stratifying patients in different categories of risk of poor outcomes.^30^ The strong correlation between LSM and HVPG has been established in several studies, showing an excellent performance of LSM also in predicting PH.^19^^,^[33], [34], [35], [36] Consequently, LSM has been officially incorporated into international guidelines for managing PH, as exemplified by the Baveno VI criteria, allowing to avoid screening endoscopy in patients with LSM <20 kPa and a platelet count >150 × 10^3^/μl, in whom it has been shown a low probability (<5%) of harbouring high-risk varices.^26^

More recently, SSM by VCTE has emerged as a complementary tool to LSM useful for predicting PH in patients with cACLD.[37], [38], [39], [40] A recent study evaluating 495 patients (90% with viral liver disease and 7% alcoholic) documented that the combination of the Baveno VI criterion with the SSM cut-off ≤46 kPa could improve diagnostic performance in excluding the presence of high-risk varices, allowing a 37% saving of EGDS.^22^ This finding has been also validated in another study using a new dedicated SSM probe (SSM@100Hz), demonstrating significant potential for reducing unnecessary endoscopic examinations. The study included 260 patients with chronic liver disease (67% viral and 30% alcoholic aetiology): the combination of the Baveno VI criterion with SSM cut-off <41.3 kPa has been shown to save 38.9% of endoscopic examinations.^21^ Similarly, the application of the proposed Baveno VII criterion (*i.e.* possibility of avoiding endoscopy in patients with cACLD of viral aetiology with LSM ≥20 kPa or platelet count ≤150 × 10^3^/μl in the presence of SSM ≤40 kPa)^25^ in our cohort of patients with PBC showed that 41.7% of EGDS would have been correctly saved, without missing high-risk varices.

To our knowledge, this is the first study in which those criteria have been applied to patients with PBC, using the new instrument with a spleen-dedicated probe (SSM@100Hz). Although this is a limited cohort, our findings provide the first proof of concept that SSM can be used for ruling out high-risk varices in patients with PBC by application of the Baveno VII criterion. In fact, none of the 19 patients with SSM ≤40 kPa who underwent EGDS had endoscopic signs of PH, regardless of LSM and platelet count. Conversely, any-size oesophageal varices were present in nine of 14 patients (64%) with SSM >40 kPa undergoing EGDS, and high-risk varices were found in three of 14 patients (21%). Nevertheless, given the limited size, our study warrants further confirmation in a larger cohort of patients.

In our cohort, the prevalence of cACLD was relatively low (17%). During a median follow-up of 15 months, 4% of patients experienced liver decompensation. Both LSM ≥10 kPa and SSM >40 kPa demonstrated excellent diagnostic performance (100% sensitivity) in predicting the risk of hepatic decompensation. Patients with LSM ≥10 kPa had a significantly higher probability of decompensating during follow-up compared with patients with LSM <10 kPa (30 *vs*. 0% at 24 months; *p* <0.0001). Similarly, patients with SSM >40 kPa had a 34% probability of hepatic decompensation at 24 months compared with 0% in those with SSM ≤40 kPa (*p* <0.0001). By combining LSM and SSM, the probability of liver decompensation was significantly higher among patients with both LSM ≥10 kPa and SSM >40 kPa: 41% at 24 months *vs*. 0% in all other groups of patients (*i.e*. LSM <10 kPa and SSM ≤40 kPa, or LSM ≥10 kPa and SSM ≤40 kPa, or LSM <10 kPa and SSM >40 kPa). These findings align with larger studies already published, reaffirming the prognostic value of LSM as a predictor of outcome in PBC,^12^^,^^16^^,^^41^ and establish the role of SSM as a complementary tool to LSM. Our data provides proof of concept that both LSM and SSM are needed for better prognostication in PBC, as the combined use of LSM and SSM yields superior outcome prediction compared with LSM alone. In our cohort of patients, liver decompensation only occurred in patients with LSM ≥10 kPa and SSM ≥40 kPa; therefore, patients with both LSM <10 kPa and SSM ≤40 kPa should be considered not at risk of developing decompensation. The combination of LSM and SSM accurately identified the ‘at-risk’ patients for developing hepatic decompensation, who may benefit from closer monitoring. Coupling LSM and SSM reduced the number of such patients from 20 of 114 (17.5%) with LSM ≥10 kPa to 14 of 114 (12.3%) when both LSM ≥10 kPa and SSM ≥40 kPa were considered, without missing any patients. Although this reduction may appear insignificant in the current cohort, it still represents a relative reduction of more than 25% and could be a significant step forward for the overall PBC population. This is clearly a clinical application for SSM coupled with LSM and, if further confirmed in larger cohorts of patients with PBC, will facilitate the implementation of a tailored approach to PBC management. The predictive value of LSM has been fully confirmed by a recent multicentric study conducted on >3,000 patients with PBC,^16^ which demonstrated that an increase in baseline LSM value from 5 to 30 kPa correlates linearly with the hazard ratio for decompensation, mortality, and transplantation, playing the most powerful prognostic tool, over UK-PBC and Globe PBC prognostic scores. Moreover, the diagnostic performance of LSM in predicting death, liver complications, and transplantation remained stable over time.^9^

To date, there are no published studies assessing the prognostic value of SSM in PBC. Our study is the first one to demonstrate its role in predicting outcomes in a PBC cohort. Furthermore, it has the unique feature of having included a group of 78 and 33 patients who underwent two and three sequential evaluations for LSM and SSM by VCTE, respectively. Although in a small sample size, it demonstrated that longitudinal changes in LSM and SSM during follow-up could anticipate disease progression or stability. For instance, a patient with an increase in LSM and SSM values over time was subsequently diagnosed with histologic progression to cirrhosis and endoscopic signs of PH. The prognostic value of dynamics of LSM has been also recently highlighted in a multicentre study on 2,244 patients with PBC, indicating that increase of LSM over time, basically yearly LSM increase, is associated with poorer clinical outcomes regardless of age and LSM values at baseline.^42^ Furthermore, in a large cohort of 2,508 patients with chronic liver disease, it was shown that repeating LSM enables an individual and updated risk assessment for decompensation and liver-related mortality.^43^

There are some limitations in the present study, including the small sample size of study population, the limited number of clinical events, and a relatively short follow-up. Moreover, we observed a quite high probability of liver decompensation (41% at 2 years) in the 14 patients with both LSM ≥10 kPa and SSM >40 kPa (high-risk group). In line with our findings, it has been recently shown that patients with PBC and elevated baseline HVPG were significantly more likely to decompensate during follow-up than those with normal portal pressure: almost 40% probability of liver decompensation was observed at 2 years in patients with HVPG >12 mmHg (high-risk group) *vs*. 2% in those with raised HVPG (>5 and ≤12 mmHg) and 0% in those with HVPG ≤5 mmHg.^32^ Baseline HVPG was an independent predictor for hepatic decompensation during follow-up. This observation strengthens the rationale of applying VCTE in PBC for both LSM and SSM, which correlate well with HVPG,^19^^,^^23^ and suggests its utility at baseline and during follow-up for risk assessment, ensuring high-risk patients receive the most appropriate management.

In conclusion, our study confirms the value of LSM and establishes the ability of SSM in combination with LSM in predicting the risk of PBC decompensation, highlighting the putative role of SSM as a complementary tool for non-invasive prognostic stratification of patients with PBC by prediction of PH. Although requiring further validation, the combination of SSM, LSM, and platelet count according to the Baveno VII criterion appears to improve the prediction of high-risk oesophageal varices in PBC.

## Financial support

The authors did not receive any financial support to produce this manuscript.

## Authors’ contributions

Had full access to all the data in the study and takes responsibility for the integrity of the data and the accuracy of the data analysis: CR. Study concept and design: CR. Acquisition of data: CR, MGC, GFM, NP, DDB, RM. Statistical analysis: CR. Study supervision: MP. Drafting of the manuscript: CR, CDB, NP, MGC, GFM. Critical revision of the manuscript for important intellectual content: all the authors.

## Data availability statement

The data that support the findings of this study are available on request from the corresponding author (CR).

## Conflicts of interest

All the authors declare no conflicts of interest.

Please refer to the accompanying ICMJE disclosure forms for further details.
