# Supplementary material for: Spleen stiffness measurement predicts decompensation and rules out high-risk oesophageal varices in primary biliary cholangitis
Source: JHEP Rep. 2023 Oct 31;6(1):100952. doi: 10.1016/j.jhepr.2023.100952 (PMC10772386; doi:10.1016/j.jhepr.2023.100952)
Supplement: Multimedia component 2 [file mmc2.docx]

**CTAT methods**

**Software**

| **Software name** | **Manufacturer** | **Version** |
| --- | --- | --- |
| SPSS Statistics | IBM Corporation, Armonk, NY | 20.0 |
| FibroScan® | Echosens, Paris, France | 630 Expert |
